# Value of thyroid transcription factor (TTF)-1 for diagnosis and prognosis of patients with locally advanced or metastatic small cell lung cancer

**DOI:** 10.1186/s13000-015-0250-z

**Published:** 2015-04-02

**Authors:** Daniel Misch, Torsten Blum, Christian Boch, Timo Weiss, Catharina Crolow, Sergej Griff, Thomas Mairinger, Torsten T Bauer, Jens Kollmeier

**Affiliations:** Department of Pneumology, Lungenklinik Heckeshorn, HELIOS Klinik Emil von Behring, Walterhöferstr. 11, 14165 Berlin, Germany; Institute of Pathology, HELIOS Klinikum Emil von Behring, Berlin, Germany

**Keywords:** Small cell lung cancer, Histology, Thyroid transcription factor, Prognosis

## Abstract

**Background:**

The aim of this study was to analyze the frequency of Thyroid Transcription Factor (TTF)-1 expression in small cell lung cancer (SCLC) and its value for the diagnosis of SCLC, the response to first line treatment as well as the prognostic impact on overall survival (OS).

**Methods:**

We analyzed a total of 294 patients (m, n = 184; f, n = 110) with SCLC (stage IIIA, n = 32; IIIB, n = 87; IV, n = 175) diagnosed in our institution between January 2005 and December 2008. Patient’s characteristics comprising age, gender, histology and first line treatment were included into the analyses. For the follow-up of patients the governmental death registrar was used. The TTF-1 immunostaining was prospectively performed. CT scans of all patients were reviewed and response to treatment was evaluated using the Response Evaluation Criteria In Solid Tumors 1.0 (RECIST) criteria.

**Results:**

A total of 221 of the 294 patients were eligible for analysis. Patients with TTF-1-positive SCLC had a median OS of 374 (95% CI 306–442) days. The OS of patients with TTF1-negative SCLC was 290 (95% CI 191–389) days, which was not significantly shorter (p = 0.254). Also stratification for tumor stage did not reveal significant difference in OS. Analyzing the disease control rate (DCR) in patients with metastatic disease (stage IV), we observed a significantly (p = 0.006) improved response to treatment in the group of patients with TTF-1-expression (DCR 86% vs. 56%). Regarding the overall response rates (ORR) in the entire population, there was no difference observed between both subgroups. (TTF-1-pos. 75.3% vs. TTF-1-neg. 71.4%; p = 0.642).

**Conclusions:**

The diagnostic information of TTF-1 in SCLC seems to be limited. TTF-1 had no prognostic value concerning OS, but may serve as a predictor for response to first line chemotherapy.

**Virtual slides:**

The virtual slide(s) for this article can be found here: http://www.diagnosticpathology.diagnomx.eu/vs/5811254651472285

## Background

The DNA-binding protein thyroid transcription factor 1 (TTF-1) is mainly expressed in the thyroid gland and the lungs and plays an important role in the development of these organs. It is expressed in type II pneumocytes and Clara cells and has a crucial part in regulating the expression of various genes, such as for the surfactant or the Clara cell protein [1-5].

It is known that the expression of TTF-1 is helpful to distinguish primary lung cancer from other non-pulmonary malignancies. The expression differs among the different histologic subtypes of lung carcinoma [6-9]. While TTF-1-expression was found to be frequent in adenocarcinoma and small cell lung cancer, it appears to be rare in patients with squamous or large cell carcinoma. In SCLC, the expression of TTF-1 was found in about 85-90% of cases [10]. Furthermore, it has been described that non-pulmonary small cell carcinoma can express TTF-1 due to their neuroendocrine differentiation [11]. Thus, the diagnostic value of TTF-1 in SCLC remains questionable. If it plays a role in the carcinogenesis of lung or thyroid cancer remains unclear, although there is evidence that it may be associated with the neuroendocrine differentiation of tumor cells [12].

Recent studies were addressed to the prognostic value of TTF-1-expression in lung carcinoma. Concerning NSCLC, TTF-1-expression is associated with better overall survival, which is even more pronounced in the subgroup of patients with adenocarcinoma [9,13-16]. A further aspect of any biomarker is its possible predictive value concerning response to chemotherapy. To our knowledge, there is currently no study which analyzed overall survival as well as response to first line chemotherapy of SCLC according to TTF-1-expression.

Therefore, the present study analyzes the frequency of TTF-1-expression in patients with locally advanced or metastatic small cell lung cancer and its prognostic value concerning overall survival as well as the predictive value for response to treatment.

## Methods

### Patient’s data and data acquisition

**Figure 1:**
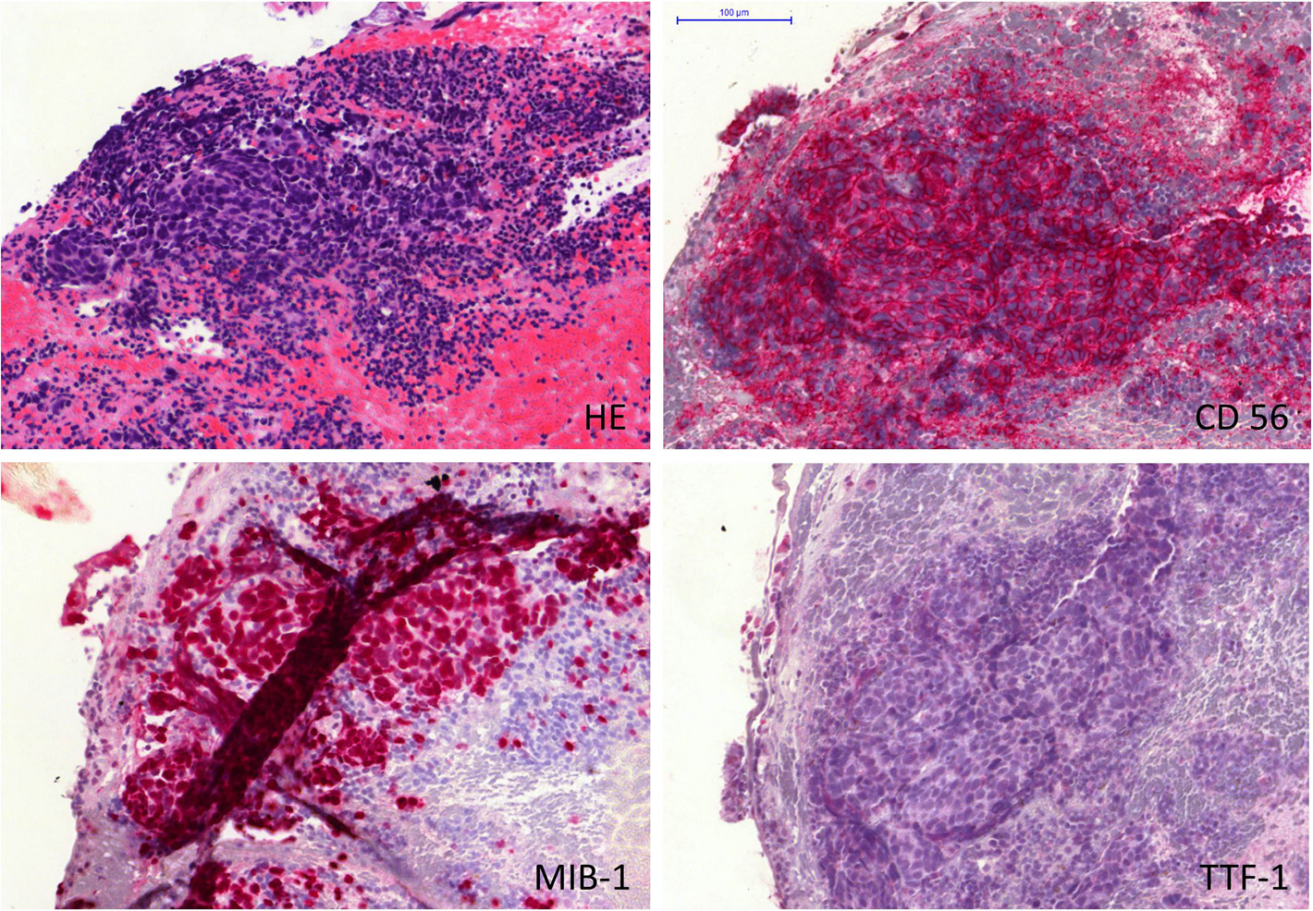
**Example of TTF-1 negative small cell lung cancer (SCLC) staining.** Left upper panel: Hematoxylin and eosin stain (HE). Right upper panel: Neural cell adhesion molecule (CD56). Left lower panel: Ki-67 Proliferation marker (Clone MIB-1). Right lower panel: Thyroid transcription factor-1 (TTF-1).

**Figure 2:**
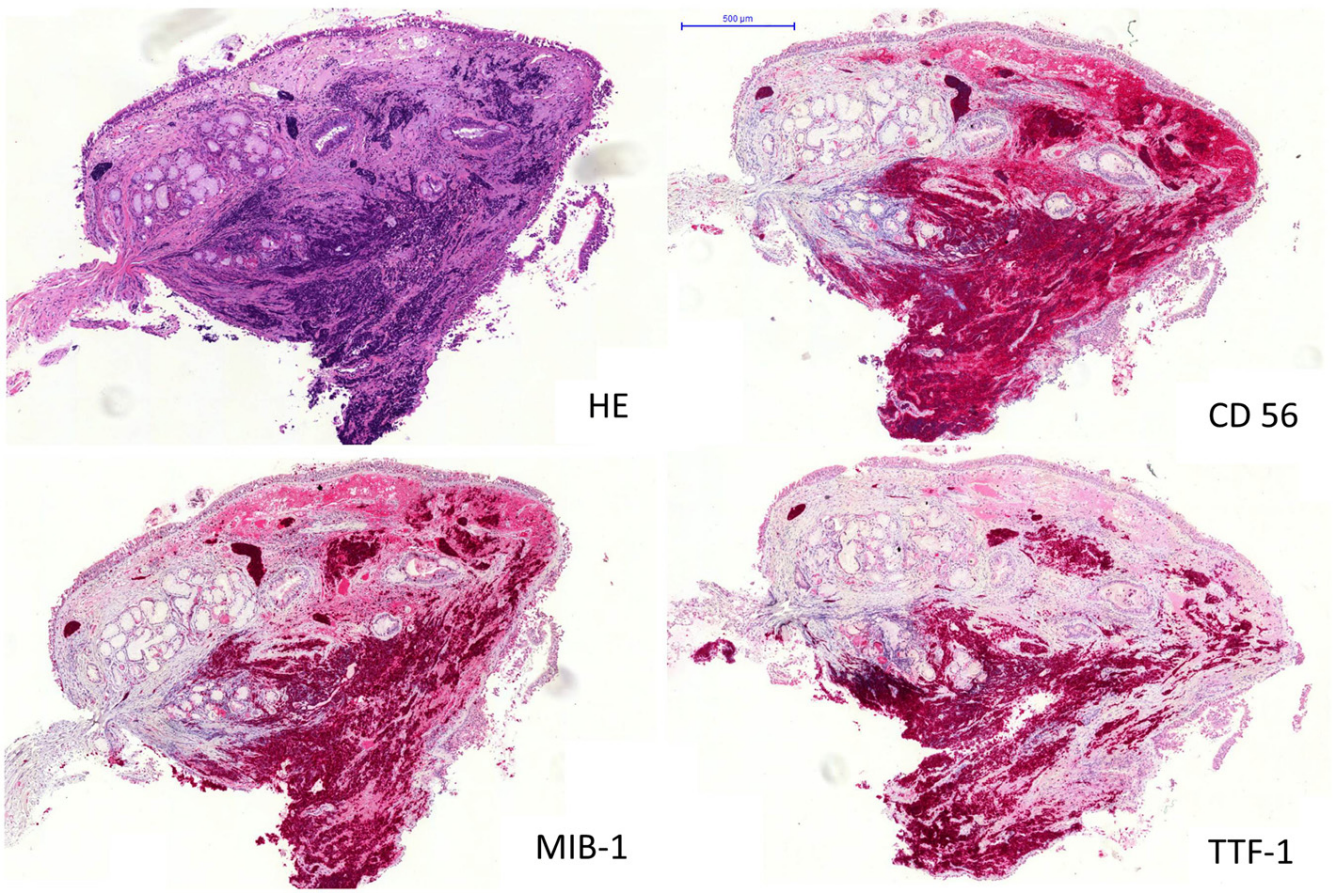
**Example of TTF-1 positive small cell lung cancer (SCLC) staining.** The tumor was identified as TTF-1 positive when more than 5% of cells stained for TTF-1. Left upper panel: Hematoxylin and eosin stain (HE). Right upper panel: Neural cell adhesion molecule (CD56). Left lower panel: Ki-67 Proliferation marker (Clone MIB-1). Right lower panel: Thyroid transcription factor-1 (TTF-1).

We retrospectively analyzed 294 patients (male, n = 184; female, n = 110) who were treated for histologically proven locally advanced or metastatic small cell lung cancer in our institution between January 2005 and December 2008. Patients characteristics were entered into a database and the following parameters were extracted for analysis: Age, gender, date of diagnosis, TNM classification (UICC 6) [17], initial therapy including chemotherapy and radiotherapy, response to first line treatment according to RECIST 1.0 [18] and the TTF-1 status of the diagnostic specimen. The TTF-1-immunostaining was performed prospectively using standard immunohistochemistry (Antibody Clone TTF-1 SP141, Ventana Medical Systems, DAB detection Kit). The immunostained cells were considered positive only when distinct nuclear staining was identified [19]. The tumor was identified as TTF-1 positive when more than 5% of cells stained for TTF-1. Further immunohistochemical markers stained were CD56, KL-1 or CKMNF-116 and MIB-1 [Figures 1 and 2].

Survival of these patients was followed up on a regular basis and mortality data were verified every six month with the governmental death registrar.

Data were gathered for internal quality control on a routine basis and all patients gave informed consent for data collection. The institutional review board (IRB-LungClinic Heckeshorn) therefore waived the need for ethical committee approval.

### Therapy

Standard therapy in our institution comprised chemotherapy, radiation and their combination. Standard chemotherapy in all stages comprised cisplatin (80 – 100 mg/m2 body surface, d1) or carboplatin (AUC 5 in cases with renal insufficiency with a glomerolar filtration rate < 60 ml/h) in combination with etoposide (100 – 140 mg/m^2^ body surface, d1-3). A combination radiochemotherapy was considered in stage III and applied simultaneously or sequentially according to the performance status of the patients. Palliative irradiation was applied to patients for the local control of tumor deposits, i.e. of bone or brain metastases. Patients diagnosed in stage IV received preferably standard palliative chemotherapy as described above. All other lines of chemotherapy were noted until the patient died or cases were censored for this analysis. The number of lines is reported per patient. According to the treating physician best supportive care was applied in patient with low functional status and/or denied informed consent for therapy.

### Response to therapy

Patients on treatment were followed-up on a regular basis, performing a chest CT and a CT of the upper abdomen (including the adrenal glands) every second cycle. The final staging for the response evaluation to first line chemotherapy was performed 3 weeks +/− 7 days after completion of first line treatment. The response was defined according to RECIST 1.0 [18]: Complete Response (CR) meaning complete disappearance of all target lesions; Partial Response (PR) meaning at least 30% reduction of the sum of all target lesions longest diameters; Progressive Disease (PD) meaning at least 20% increase of the sum of all target lesions longest diameters, taking as reference the smallest measurements recorded since the treatment started and Stable Disease (SD) being defined as neither sufficient shrinkage to qualify for PR nor sufficient decrease to qualify for PD.

The disease control rate (DCR) was defined as all patients showing at least a stable disease after first line treatment whereas the overall response rate comprised only patients with a complete or partial response.

#### Statistical analysis

All frequencies are reported as number and percentage. Continuous variables are reported as mean ± standard deviation. Frequencies were analyzed with Chi-square statistics with Fisher’s exact test were needed. The disease control rate (DCR) was analyzed according to the effects of stage (III vs. IV) and TTF-1-status with a Chi-square test with layer analysis (Mantel-Haenszel statistics). Continuous survival was analyzed with Kaplan-Meier statistics and strata were compared with log-rank analyses. Survival time is reported as median days and the 95% confidence interval. All analyses were carried out with the Statistical package for Social Sciences (SPSS® Version 19.0) on a Microsoft Windows® operating system.

## Results

Mean age of all patients was 64.1 ± 10.2 years (n = 294). A total of 119/294 patients (40.5%) had stage III (IIIA, n = 32, 10.9%; IIIB, n = 87, 29.6%) and 175/294 patients (59.5%) had stage IV SCLC according to the UICC-6 staging system.

Of the patients with stage III disease (n = 119/294, 40.5%), 70/119 (58.8%) received combined radio- and chemotherapy (simultaneous n = 50, 42.0%; sequential, n = 20, 16.8%; all platinum-based) as first line therapy, whereas 6/119 patients (5.0%) had chemotherapy with palliative radiotherapy (i.e. incomplete dose) only. 30/119 patients (25.2%) received only chemotherapy and 8/119 (6.7%) only radiotherapy (definitive n = 3, 2.5%; palliative n = 5, 4.2%). Best supportive care was applied in 4/119 cases (3.4%) and 1/119 therapies could not be retrieved. A total of 30/119 patients (25.2%) had prophylactic brain irradiation followed by of systemic treatment.

In the population of the patients with stage IV (n = 175/294, 59.5%) small cell lung cancer, 160/175 (91.4%) received palliative chemotherapy (platinum-based, n = 154/160, 96%) for systemic treatment. Out of these, 52/160 patients (32.5%) additionally received palliative radiotherapy due to brain metastases (n = 24), bone metastases (n = 12) or for local control of thoracic tumor sites (n = 21). After palliative chemotherapy, 9/175 patients (5.1%) had prophylactic brain irradiation. 7/175 patients (4.0%) received only palliative radiotherapy. Best supportive care was administered in 4/175 patients (2.3%). No information on therapeutic regime was available for 2/175 cases (1.2%).

TTF-1 immunhistochemistry was available for 221/294 patients (75.2%) of the entire population. Of these, 38/221 (17.2%) had TTF-1 negative small cell lung cancer, whereas 183/221 (82.8%) tumors showed reactivity in TTF-1 immunostaining. The distribution of gender was equal in both groups. Comparing the percentage of TTF-1 negative tumors according to stage, no significant difference was observed [Table 1].

**Table 1:** **Patient characteristics**

	**all**	**TTF1 +**	**TTF1 -**	**p-value**
**Entire population**	221	183/221 (82.8%)	38/221 (17.2%)	
**Age (mean ± SD)**	64.7 ± 10.2	64.0 ± 10.2	68.1 ± 9.6	0.023*
**Female n (%)**	76/221 (34.4)	63/183 (34.4%)	13/38 (34.2%)	1.000**
**Male n (%)**	145/221 (66.6)	120/183 (65.6%)	25/38 (65.8%)	
**Stage IIIA n (%)**	21/221 (9.5)	16/183 (8.7%)	5/38 (13.2%)	
**Stage IIIB n (%)**	64/221 (29.0)	56/183 (30.6%)	8/38 (21.0%)	
**Stage IV n (%)**	136/221 (61.5)	111/183 (60.7%)	25/38 (65.8%)	0.412**

Overview of the proportion of patients with TTF1-positive and TTF1-negative SCLC in the entire patient population and in the different stage groups.

*Student’s T-Test.

**Chi-Square Test.

Of the patients included into the per protocol analyses 181/221 (81.9%) died during the observation period. The median overall survival of all patients with known TTF-1-status was 341 days (95% CI 282–400 days). Patients with stage IV disease showed a significant shorter overall survival (290 [95% CI 249–331] days) as compared to those with stage IIIA (488 [95% CI 352–624] days) and IIIB disease (451 [95% CI 354–548] days; p < 0.001 log rank analysis for all strata).

**Figure 3:**
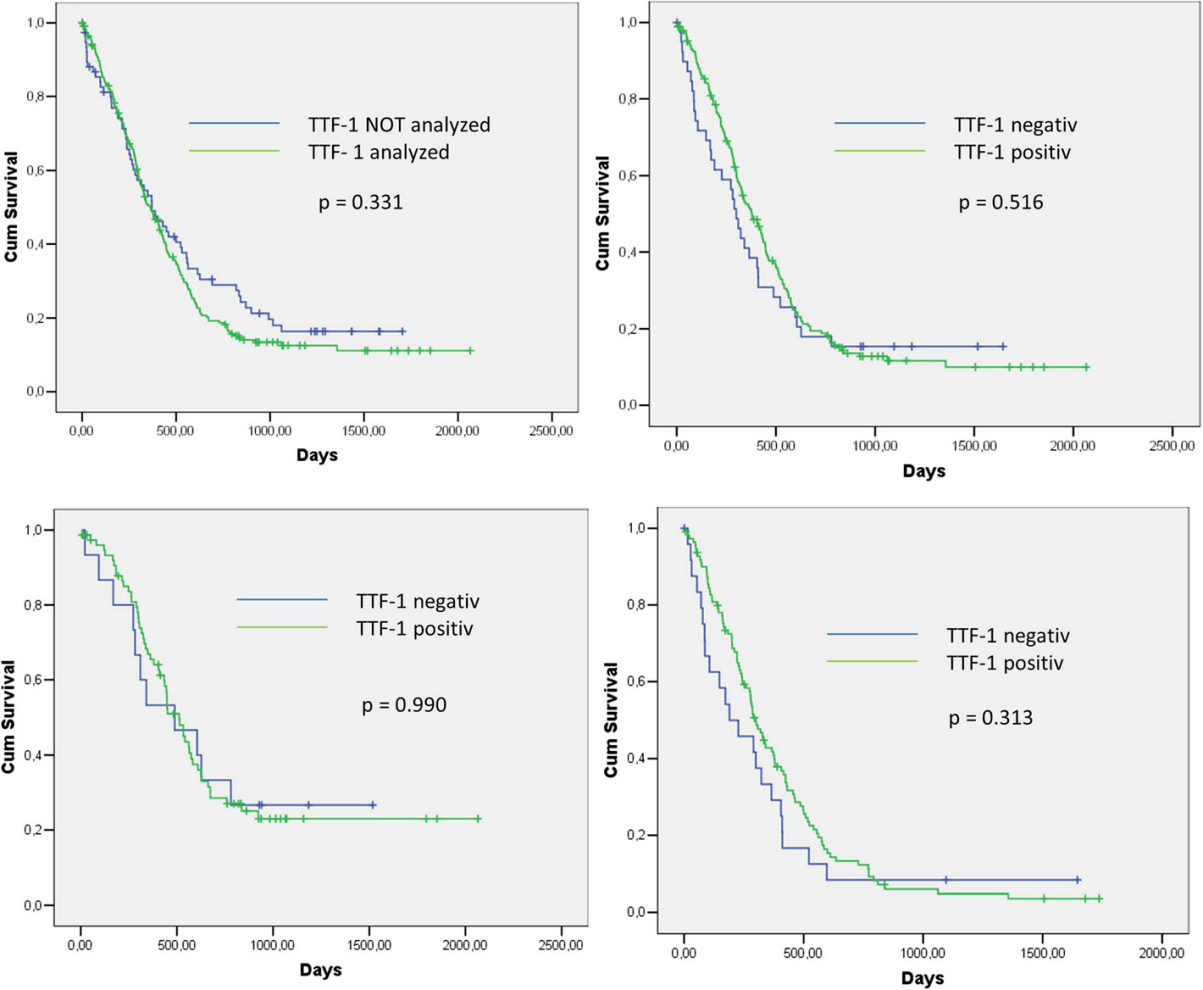
**Survival Analysis and sub-analysis for disease stage according to TTF-1 (Kaplan-Meier plots with log-rank analysis; PP = per protocol, ITT = intention to treat).** Left upper panel: ITT (n = 294) vs. PP population (n = 221; p = 0.331). Right upper panel: PP population: TTF-1 positive (n = 183) vs. TTF-1 negative patients (n = 38; p = 0.516). Left lower panel: PP sub-population with non-metastatic disease (n = 85): TTF-1 positive (n = 72) vs. TTF-1 negative patients (n = 13; p = 0.990). Right lower panel: PP sub-population with metastatic disease (n = 136): TTF-1 positive (n =111) vs. TTF-1 negative patients (n = 25; p = 0.313).

Stratifying for the TTF-1-status, median overall survival of all patients with TTF-1 positive tumors was 374 (95% CI 306–442) days. Patients with TTF-1 negative SCLC had a median overall survival of 290 (95% CI 191–389) days, which was not significantly different from those with TTF-1 positive disease (p = 0.254). When analyzing the overall survival according to TTF-1 for the different stage groups, no significant difference was observed for stage IV (p = 0.237) as well as for IIIA (p = 0.852) and IIIB patients (p = 0.506) [Table 2 and Figure 3].

**Table 2:** **Overall survival (OS) in days according to TTF1 in the entire population and in the subgroups of patients with stage IIIA, IIIB and IV disease**

	**TTF1 +**	**TTF1 -**	p-value*
**Entire population**	374 (306–441)	290 (191–389)	0.254
**Stage IIIA**	533 (361–704)	488 (172–803)	0.852
**Stage IIIB**	512 (413–610)	282 (87–477)	0.506
**Stage IV**	302 (253–351)	227 (58–396)	0.273

*log-rank analyses.

For the analysis of response to treatment according to TTF-1-status of the tumor, patients were excluded when no therapy or best supportive care (n = 18) was applied or response to treatment was not available (n = 25). Therefore, 178/221 (77.1%) of all patients with known TTF-1-expression were eligible. Of these, 28/178 patients (15.7%) had TTF-1 negative SCLC.

Analyzing univariately the disease control rate (DCR), we observed a significantly (p = 0.013) improved response to treatment in the group of patients with TTF-1-expression (DCR 135/150 (90%), CR, n = 8; PR, n = 105; SD, n = 22; PD, n = 15) as compared to those without TTF-1-expression (DCR 20/28 (71.4%); CR, n = 4; PR, n = 16; SD, n = 0; PD, n = 8). Regarding the overall response rates (ORR) in the entire population of stage III and IV patients, the number of patients showing at least a partial remission was only slightly different between both groups (TTF-1-pos. 75.3% vs. TTF-1-neg. 71.4%) failing to reach the level of significance (p = 0.642) [Table 3].

**Table 3:** **Response to treatment according to TTF1-expression (n = 178)**

	**TTF1 + 150 (84.3%)**	**TTF1 –n = 28 (15.7%)**	**p-value**
**CR**	8 (5.3%)	4 (14.3%)	
**PR**	105 (70.0%)	16 (57.1%)	
**SD**	22 (14.7%)	0	
**PD**	15 (10.0%)	8 (28.6%)	**0.003**
**RR**	75.3%	71.4%	0.642
**DCR (overall)**	135 (90.0%)	20 (71.4%)	**0.013**
**DCR stage III**	56/58 (96.6%)	10/10 (100%)	0.726
**DCR stage IV**	79/92 (85.9%)	10/18 (55.6%)	**0.006**

Overall response rate (RR) is defined as the proportion of patients having at least a partial response according to RECIST1.0. Disease control rate (DCR) is defined as all patients with at least a stable disease according to RECIST1.0. (CR = complete response; PR = partial response; SD = stable disease; PD = progressive disease).

When the DCR was analyzed separately for non-metastatic (stage III) and metastatic patients (stage IV), it was significantly higher in patients with TTF-1-positive SCLC in stage IV (79/92, 85.9%) compared to patients in the similar stage but with TTF-1-negative carcinoma (10/18, 55.6%; p = 0.006). This analysis did not show any difference in stage III patients because the DCR was very high for both groups of patients (DCR TTF-1-pos. 56/58 96% versus TTF-1-neg. 10/10 100%; p = 0.726).

## Discussion

TTF-1 is an immunhistochemical marker, which can help to differentiate between primary lung carcinoma and non-pulmonary cancer. In NSCLC, TTF-1-expression occurs mainly in adenocarcinoma and plays a crucial role in differentiating primary lung adenocarcinoma from pulmonary metastatic disease [20]. In SCLC, the expression of TTF-1 was found to be very frequent in various studies. However, a review of the literature reveals that SCLC show a lack of TTF-1 expression in about 10–15% of all cases ([6,7,21-25]). This is in line with the results of our study, where 17% of SCLC did not show TTF-1-expression. The frequency was neither associated with disease stage (III or IV), age nor gender.

Various studies investigated the immunhistochemical expression pattern of non-pulmonary small cell carcinoma, such as prostate or gastrointestinal SCC, revealing discrepant results. While Ordanez et al. found TTF-1-expression in only 4 of 54 cases, another study by Yao et al. on 18 patients with small cell prostate carcinoma showed TTF-1-expression in 83% of cases [10,11]. This indicates that TTF-1 is not an exclusively pulmonological marker but rather points towards a neuroendocrine origin of the neoplastic cell [1,12]. This is strengthened by the fact that non-pulmonary SCC as well as SCLC express other neuroendocrine markers such as chromogranin A, neuro-specific enolase (NSE), CD57 or CD56 [26-31]. Therefore, the value of TTF-1 for origin-diagnosis of small cell carcinoma seems to be dispensable, especially as the therapeutic approaches in cases of SCC do usually not differ according to the origin if the primary tumor site.

Another aspect of TTF-1-expression is a potentially possible predictive value on overall survival of lung cancer patients. For NSCLC, there seems to be evidence that patients with TTF-1 positive tumors show improved overall survival compared to those with TTF-1 negative tumors [9,13-16,23]. In our survival analyses including 221 SCLC patients, no significant difference could be shown for the comparison between patients with different TTF-1-expression. After stratifying for stage of disease, the results persisted in the subgroups of patients with stage III as well as stage IV disease.

Regarding the response to therapy according to RECIST, the response rates of first line chemotherapy in the population of SCLC patients are known to be high in contrast to NSCLC. However, a number of approximately 10-20% of all SCLC does not respond to first line chemotherapy. The reason for that is mainly unknown. As the chemotherapy is known to cause significant side effects decreasing the quality of life of patients, it is of essential need to identify markers that may predict response to chemotherapeutic treatment. To our knowledge, there is currently no other study addressed to the possible implication of TTF-1-expression on response to chemotherapy in SCLC. In our analysis, a significantly higher disease control rate (DCR) in the group of TTF-1-positive SCLC could be shown, assuming a higher chemosensitivity of these tumors. Stratified by stage, this effect was highly significant in stage IV but could not be reproduced in stage III. We see this as a result of the high radiation rate in the stage III subgroup (overall 70.5%), masking the presumed lower chemosensitivity of TTF-1-negative tumors.

Although this was a strong effect in metastatic disease, this difference did not result in a relevant overall survival benefit. Furthermore, response rates are still high in the subgroup of TTF-1-negative patients (>55%), which does not allow a reliable identification of patients not benefiting from platinum based chemotherapy with TTF-1 only. If TTF-1 could be of predictive value used side-by-side with other biomarkers (i.e. MIB-1) should be investigated in further studies.

## Conclusions

In summary, the diagnostic value of routine immunhistochemistry of TTF-1 in case of suspected SCLC appears to be low. Concerning the diagnostic aspect, the morphology of tumor cells and other immunhistochemical markers, such as chromogranin A, neuro-specific enolase (NSE), CD57 or CD56, are sufficient to enable the diagnosis of small cell cancer with neuroendocrine differentiation. As TTF-1 can be missing in case of SCLC and as well be present in case of non-pulmonary SCC, it seems to be of limited value for the definition of the primary tumor.

Concerning the prognostic aspect, our data indicate no prognostic implication of TTF-1-expression in SCLC patients. However, we observed a strong association between the absence of TTF-1-expression and the risk of first line failure in patients receiving platinum based chemotherapy only.
